# A gut microbiota–metabolite–host gene score for subtyping and predicting the treatment response in rheumatoid arthritis: integrative analysis with in silico knockout validation

**DOI:** 10.1186/s40643-026-01149-7

**Published:** 2026-10-20

**Authors:** Xiang-Fei Sun, Yi-Jie Yang, Chen-Long Li, Tai Huang, Qi Wang, Qi Yu

**Affiliations:** School of Management, Shanxi Medical University, Taiyuan, China

**Keywords:** Rheumatoid arthritis, Gut microbiota–metabolite–host gene axis, GMS, In silico knockout

## Abstract

**Background:**

The pathogenesis of rheumatoid arthritis (RA) involves a complex interplay between immune dysregulation and metabolic disturbance, with the gut microbiota and its metabolites playing pivotal roles. However, integrated biomarkers based on the “gut microbiota–metabolite–host gene” regulatory axis that can quantify its impact on RA stratification and therapeutic response are lacking.

**Methods:**

We integrated multiple RA transcriptomic datasets to screen for host genes explicitly associated with the gut microbiota and metabolites using the gutMGene database. A gut microbiota–metabolite–host score (GMS) was constructed via principal component analysis (PCA). Immune infiltration patterns were evaluated using CIBERSORT. Furthermore, scRNA-seq was employed to map the cellular origins of key genes, and in silico knockout analysis using scTenifoldKnk was performed to simulate the functional ablation of core host genes within this regulatory axis. Additionally, molecular docking was conducted to validate the binding affinities between the key host proteins and the optimal small molecule.

**Results:**

We identified seven microbiota–metabolite–host hub genes (FOS, JUN, IDO1, PEMT, EGFR, ABCC4, and FADS3) and constructed the GMS. The GMS stratified patients with RA into two distinct functional subtypes: an adaptive immune activation subtype and an inflammatory–metabolic composite subtype. Notably, the GMS demonstrated superior performance in predicting responses to anti-TNF and rituximab therapies. The scRNA-seq analysis revealed high expression of FOS and JUN in T cells and NK cells. In silico knockout analysis further revealed the central transcription factors FOS and JUN as key executors of this axis, as their ablation significantly affected osteoclast differentiation and the enrichment of TNF/MAPK inflammatory pathways. These computational findings support the core mechanism by which gut microbiota–metabolite signalling drives RA joint pathology via FOS/JUN. Molecular docking results confirmed strong binding interactions between the identified active molecules and their respective target proteins.

**Conclusion:**

This study establishes a GMS based on the gut microbiota–metabolite–host gene regulatory axis as a robust tool for molecular subtyping and predicting biological therapy responses in patients with RA. Crucially, through integrated computational modelling, including in silico knockout and molecular docking validation, we identified FOS/JUN as critical executor genes linking the gut microenvironment to RA bone destruction, providing novel targets for therapeutic strategies aimed at the gut–joint axis.

**Graphical abstract:**

**Figure:**
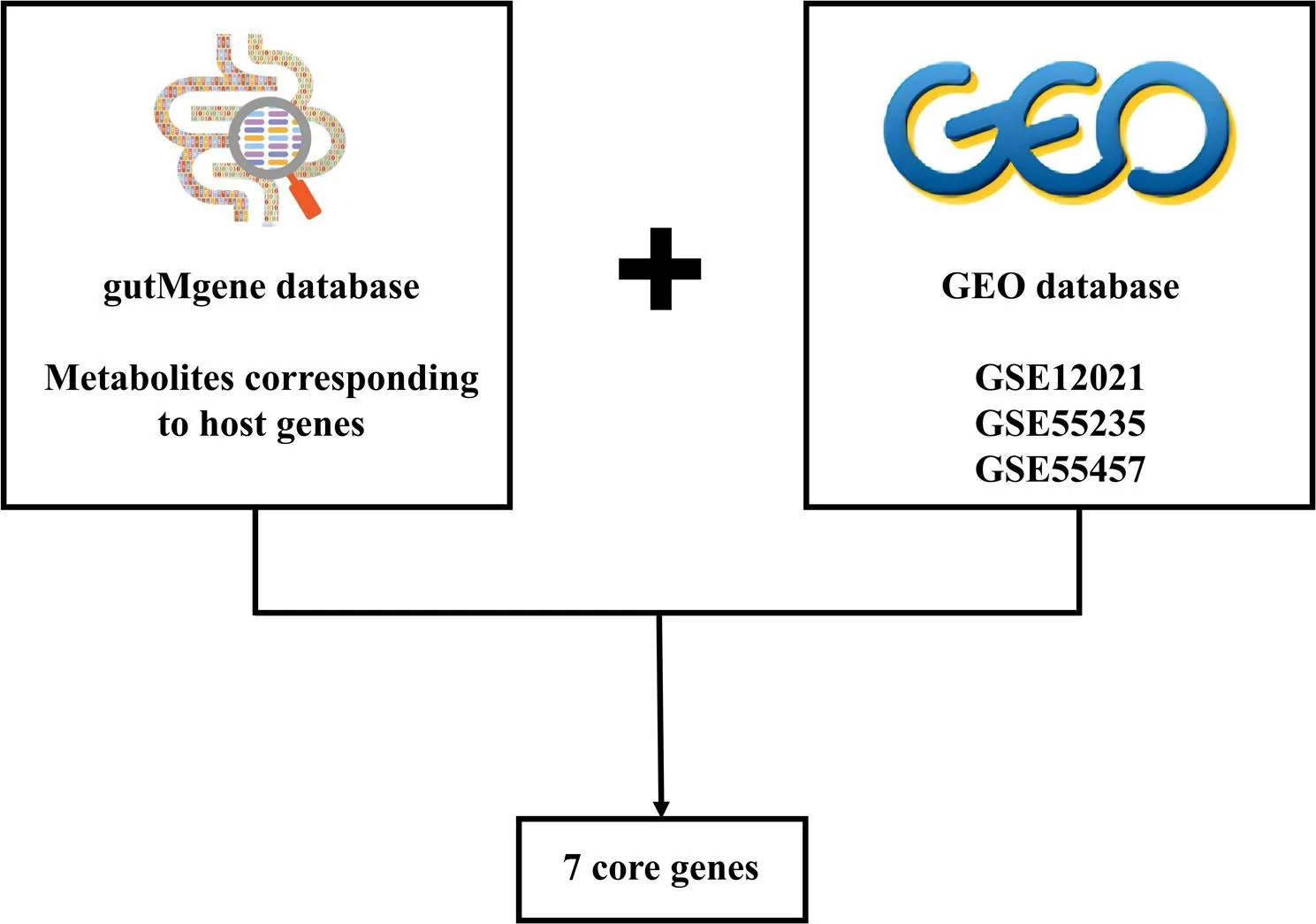

## Introduction

Rheumatoid arthritis (RA) is a systemic autoimmune disease driven by a complex interplay of genetic, environmental, and hormonal factors, manifesting as synovial inflammation and hyperplasia that result in the irreversible destruction of cartilage and bone (Marty et al. 2001). Despite the advent of biological and targeted synthetic disease-modifying antirheumatic drugs (DMARDs), which have significantly improved prognoses alongside conventional therapies (Yang et al. 2017), critical hurdles remain. Foremost among these is the marked interindividual heterogeneity in response to biological agents, such as anti-TNF inhibitors and rituximab (Nam et al. 2010). Moreover, reliable biomarkers for precise molecular subtyping and treatment response prediction remain elusive. Consequently, deciphering the intricate pathophysiology of RA and constructing molecular mechanism-based predictive models are pivotal steps towards realising precision medicine.

In recent years, the “gut–joint axis” hypothesis has offered a novel paradigm for elucidating the pathogenesis of RA (Chasov et al. 2024). Accumulating evidence suggests that the gut microbiota, which acts as a central hub for host–environment interactions, is intimately linked to the onset and progression of RA via structural and functional disturbances, collectively termed dysbiosis. In addition to directly participating in RA pathogenesis through immune regulation and compromised gut permeability (Pianta et al. 2017; Scher and Abramson 2011), the gut microbiota plays a critical role by releasing metabolites, such as short-chain fatty acids (SCFAs) and tryptophan derivatives (Zeng et al. 2025). These metabolites act as signalling molecules to remotely orchestrate host immune homeostasis and joint inflammation via the circulatory system. Consequently, this “microbiota–metabolite–host gene” cascade serves as a pivotal bridge connecting environmental factors with host immune responses.

However, current research still faces significant challenges. First, most studies are confined to single-omics analyses (Kapoor and Gulati 2025; Yu et al. 2022), and there is a lack of systematic investigations that integrate the “gut microbiota–metabolite–host target gene” axis into a functional regulatory framework, making it difficult to quantify the specific contribution of this axis to RA phenotypes. Second, although genome-wide association studies have identified numerous susceptibility loci for RA (Saevarsdottir et al. 2022), the specific effector genes that are directly regulated by gut microbiota-derived metabolites remain unclear, and the gene–environment interaction mechanisms between these two layers have yet to be elucidated. Finally, existing biomarkers predominantly focus on single dimensions (Liang et al. 2025), and there remains a paucity of integrated multi-omics scoring systems that combine the gut microecological status with host immune–metabolic characteristics, thereby limiting more precise clinical subtyping and individualised treatment guidance.

To address these challenges, this study aims to construct a comprehensive GMS by integrating multi-omics data to bridge the gap between gut microecology and host immune responses in RA. We systematically identified key hub genes regulated by the microbiome and established a robust scoring system for molecular subtyping and therapeutic response prediction. Furthermore, we leveraged scRNA-seq and in silico knockout simulations to validate the functional roles of these genes, specifically by elucidating how the gut–joint axis drives joint pathology via FOS/JUN signalling. Our findings provide a novel tool for precision medicine in patients with RA.

## Materials and methods

### Data acquisition and preprocessing

To identify pathogenic genes and evaluate RA therapies, three bulk transcriptomic datasets (GSE12021, GSE55235, and GSE55457) from patients with RA and healthy controls were used for differential expression analysis (DEGs) and hub gene identification. Two independent datasets (GSE37107 and GSE15258) were utilised to validate the predictive ability of rituximab and anti-TNF therapeutic responses. The peripheral blood single-cell dataset GSE159117 was used to characterise cellular landscapes and validate the hub genes. A gut microbiome-related host gene set was retrieved from gutMGene (Qi et al. 2025). All bulk data were normalised and preprocessed to ensure comparability across platforms. Detailed information regarding the data used in this study is presented in Table 1.

**Table 1:** Summary information of patients with RA

Dataset	Subjects	Experiment type	Platforms	Collection site	Reference
Discovery cohort					
Accession: GSE12021	12 RA vs. 9 HC	Expression profiling by array	GPL96\GPL97	Hannover, Germany	PMID: 18,721,452
Accession: GSE55235	10 RA vs. 10 HC	Expression profiling by array	GPL96	Berlin, Germany	PMID: 24,690,414
Accession: GSE55457	13 RA vs. 10 HC	Expression profiling by array	GPL96	Eisenberg, Germany	PMID: 24,690,414
Treatment cohort					
Accession: GSE15258	Anti-TNF (53 Response vs. 22 Nonresponse)	Expression profiling by array	GPL570	Cambridge, USA	PMID: 19,699,293
Accession: GSE37107	Rituximab (8 Response vs. 6 Nonresponse)	Expression profiling by array	GPL6947	Amsterdam, Netherlands	PMID: 22,540,992
single-cell cohort					
Accession: GSE159117	Peripheral blood mononuclear cells of patients with RA	Expression profiling by high throughput sequencing	GPL23227	Beijing, China	PMID: 35,015,026

### Identification of DEGs and hub genes

DEGs between the RA and control groups were identified using the limma package, with statistical significance defined as an adj. *P* < 0.05 and |log_2_FC|>1. To identify robust hub genes potentially regulated by microbiota metabolites, we intersected the DEGs from the three discovery datasets with the microbial metabolite-related gene set from gutMGene using Venn diagram analysis. The expression levels of the DEGs were visualised using box plots and volcano plots. A chord diagram was constructed to illustrate the correlation network among these hub genes.

### Construction of the core gene‒metabolite‒microbiome interaction network

To elucidate the regulatory mechanisms linking the identified hub genes with the gut microenvironment, we utilised the gutMGene database to determine interactions between host genes, the gut microbiota, and metabolites.

### Construction of the GMS

To quantify the characteristics of gut microbiome-related genes in patients with RA, we constructed a GMS based on hub genes. First, the expression matrix of these genes in the RA samples was extracted and subjected to Z-score normalisation. PCA was subsequently employed for dimensionality reduction, where the loading values of the first principal component (PC1) served as the weights for each gene in the scoring model. Finally, the GMS for each sample was calculated by summing the product of the normalised expression value of each gene and its corresponding weight. On the basis of the median GMS, the patient cohort was stratified into a high-score group and a low-score group. The GMS for the treatment-response datasets was calculated using the gene weights derived from PC1.

### Functional enrichment analysis

To elucidate the biological characteristics of the two subtypes, DEGs between the two subtypes were first identified using the limma package. Genes with an adj. *P* < 0.05 and |log_2_FC|>1 were considered to indicate significant differential expression. Gene Ontology (GO) and Kyoto Encyclopedia of Genes and Genomes (KEGG) pathway enrichment analyses were subsequently performed on the identified DEGs. Functional enrichment analyses were conducted using the clusterProfiler R package, with statistical significance set at *p* < 0.05, and multiple testing correction was performed using the Benjamini‒Hochberg method to control the false discovery rate (FDR). The enrichment results were visualised using the enrichplot and ggplot2 packages.

### Immune infiltration analysis

The CIBERSORT algorithm was employed to estimate the relative proportions of 22 immune cell types (Chen et al. 2018). To explore the interactions between the seven hub genes and the immune microenvironment, the linkET package in R was used to conduct a Mantel test, which statistically evaluates the association between the gene expression profiles and the infiltration levels of various immune cell subtypes.

### Evaluation of the therapeutic response of the GMS

To assess the predictive value of the GMS for therapeutic efficacy, we analysed its distribution in patients with RA treated with anti-TNF and rituximab therapies. Furthermore, receiver operating characteristic (ROC) curve analysis was performed using the pROC package to evaluate the predictive accuracy of both the seven hub genes and the GMS. The area under the curve (AUC) was calculated to quantify the performance of these biomarkers in predicting treatment response.

### ScRNA-seq data processing

Raw gene expression matrices were processed using the Seurat R package (v4.0). To ensure data quality, cells were filtered on the basis of the following criteria: (1) cells expressing fewer than 200 or more than 4,000 genes were excluded; (2) cells with a mitochondrial gene percentage exceeding 25% were discarded to eliminate low-quality cells or potential doublets. Additionally, doublet detection was performed using scDblFinder, and predicted doublets were removed from the downstream analysis.

The remaining high-quality cells were normalised and scaled using the SCTransform method, which applies a regularized negative binomial regression to stabilise variances across the dynamic range of expression. PCA was subsequently performed on the scaled data to reduce dimensionality. The significant principal components were selected for downstream analysis on the basis of the Elbow plot and JackStraw procedures. Finally, the cells were visualised in a two-dimensional space using t-distributed stochastic neighbour embedding (t-SNE). Unsupervised clustering was conducted using the shared nearest neighbour (SNN) graph-based clustering algorithm to identify distinct cell populations. Cell types were annotated on the basis of the expression of canonical marker genes.

### In silico knockout

To predict the regulatory functions of specific genes, we performed a virtual gene knockout analysis using the scTenifoldKnk R package (Osorio et al. 2022). A raw UMI count matrix was extracted from the Seurat object of our scRNA-seq data as input. The algorithm involves constructing multiple gene coexpression networks from bootstrapped cell subsets, integrating them into a three-dimensional tensor, and then removing the target gene from the tensor space to model the transcriptional perturbation. Differentially expressed genes between the original and knockout networks were identified on the basis of changes in the gene expression trajectory using Mahalanobis distance testing. The analysis was performed using default parameters unless otherwise specified. The resulting DEGs were considered potential downstream targets affected.

### Molecular docking visualisation

Three-dimensional (3D) structural models of the proteins encoded by the ABCC4, IDO1, EGFR, and PEMT genes were retrieved and downloaded from the UniProt (https://www.uniprot.org/) and RCSB PDB (https://www.rcsb.org/) databases and supplemented with data from the AlphaFold database (https://alphafold.ebi.ac.uk/). The inclusion criteria included cocrystal structures determined by X-ray diffraction with a resolution better than 2.50 Å for use in molecular docking. The 3D structures of all traditional Chinese medicines and their active molecules were downloaded from the TCMSP database (https://old.tcmsp-e.com/tcmsp.php), where active molecules were required to have oral bioavailability (OB) greater than 30% and drug likeness (DL) greater than 0.18.

The 3D structures of the receptors and ligands were preprocessed using AutoDockTools (ADT) by removing water molecules, adding hydrogen atoms, computing charges, and selecting torsional centres and rotatable bonds (for small molecules) and were then saved in the pdbqt format for docking. The size and coordinates of the docking box were subsequently set using the grid box function in ADT. In this study, high-throughput molecular docking calculations were conducted using the Python programming language and the Qvina2 program. PyMOL software was used to visualise the intermolecular interactions between the receptors and ligands.

## Results

### Identification of microbiota-metabolite-host hub genes in RA

**Fig. 1:**
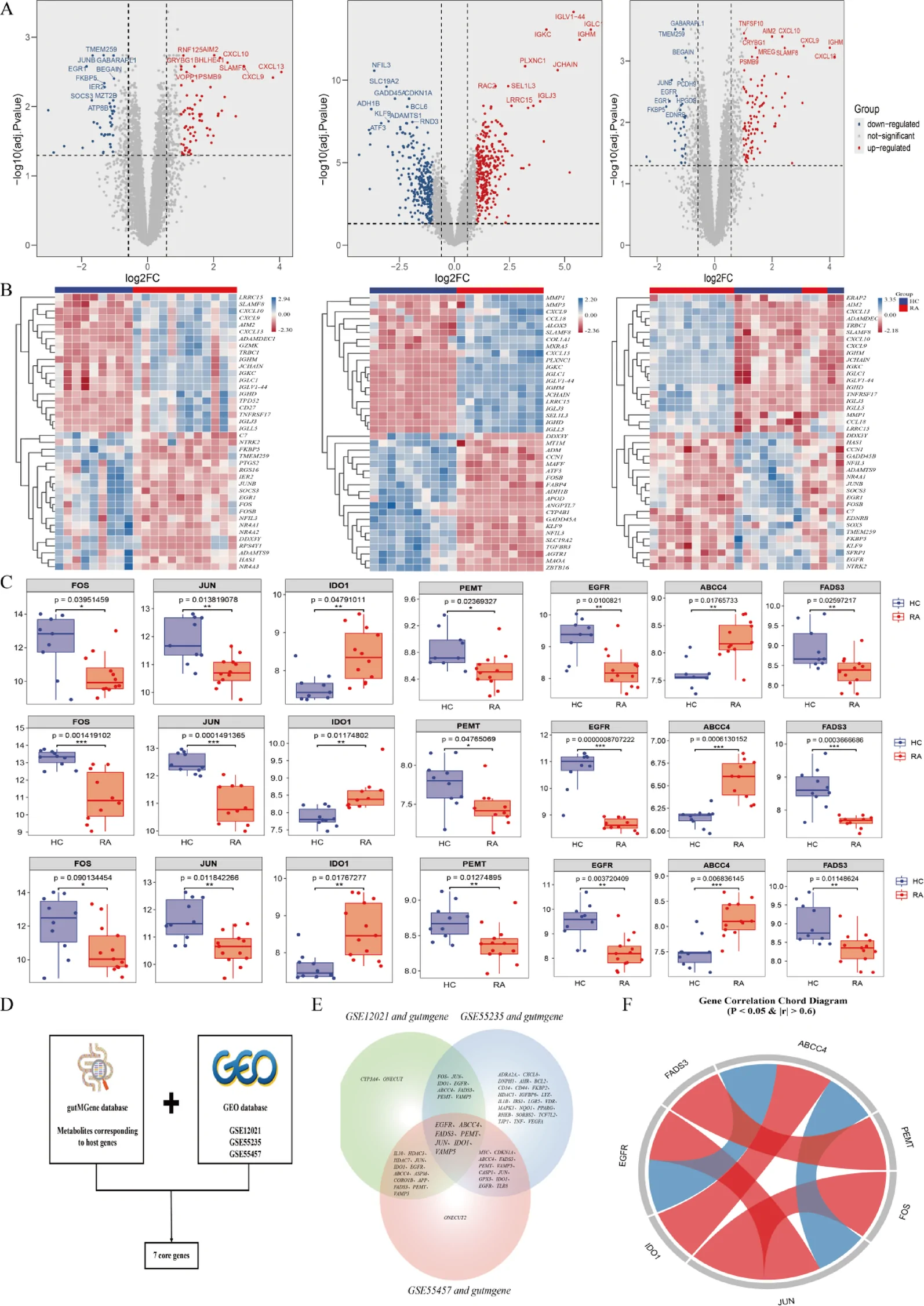
Identification of microbiota-metabolite-host hub genes in RA. **A** Volcano plots showing the differential gene expression profiles between RA patients and healthy controls in the GSE12021, GSE55235, and GSE55457 datasets. **B** Hierarchical clustering heatmaps of the DEGs in the three datasets. **C** Box plots comparing the expression levels of the 7 identified hub genes between the RA and control groups across the three datasets. **D** Schematic workflow of the study design. **E** Venn diagrams illustrating the intersection of DEGs from the GSE12021, GSE55235, and GSE55457 datasets with the gutMGene gene set. **F** Chord diagram displaying the correlation network among the seven hub genes. Red ribbons indicate positive correlations, while blue ribbons indicate negative correlations

To identify key pathogenic genes in RA, we integrated and analysed three independent datasets. Volcano plots illustrated the top 10 most significantly up- and downregulated genes in each dataset (Fig. 1A). Furthermore, hierarchical clustering heatmaps based on these DEGs clearly distinguished the RA samples from the healthy control samples, indicating robust intergroup heterogeneity (Fig. 1B).

By intersecting the DEGs from the three datasets with the host regulatory gene set from the gutMGene database, we identified seven overlapping hub genes: FOS, JUN, IDO1, PEMT, EGFR, ABCC4, and FADS3 (Fig. 1D and E). Expression analysis across the datasets revealed that, compared with those in healthy controls, the expression levels of FOS, JUN, PEMT, EGFR, and FADS3 were significantly lower in the RA group, whereas the expression levels of IDO1 and ABCC4 were significantly greater (*P* < 0.05; Fig. 1C). Additionally, a chord diagram revealed a complex correlation network among these seven hub genes. Notably, FOS and JUN were strongly positively correlated, suggesting that these genes may function synergistically to respond to microbiota metabolic signals during RA pathogenesis (Fig. 1F).

### Construction of the core gene‒metabolite‒microbiome interaction network

**Fig. 2:**
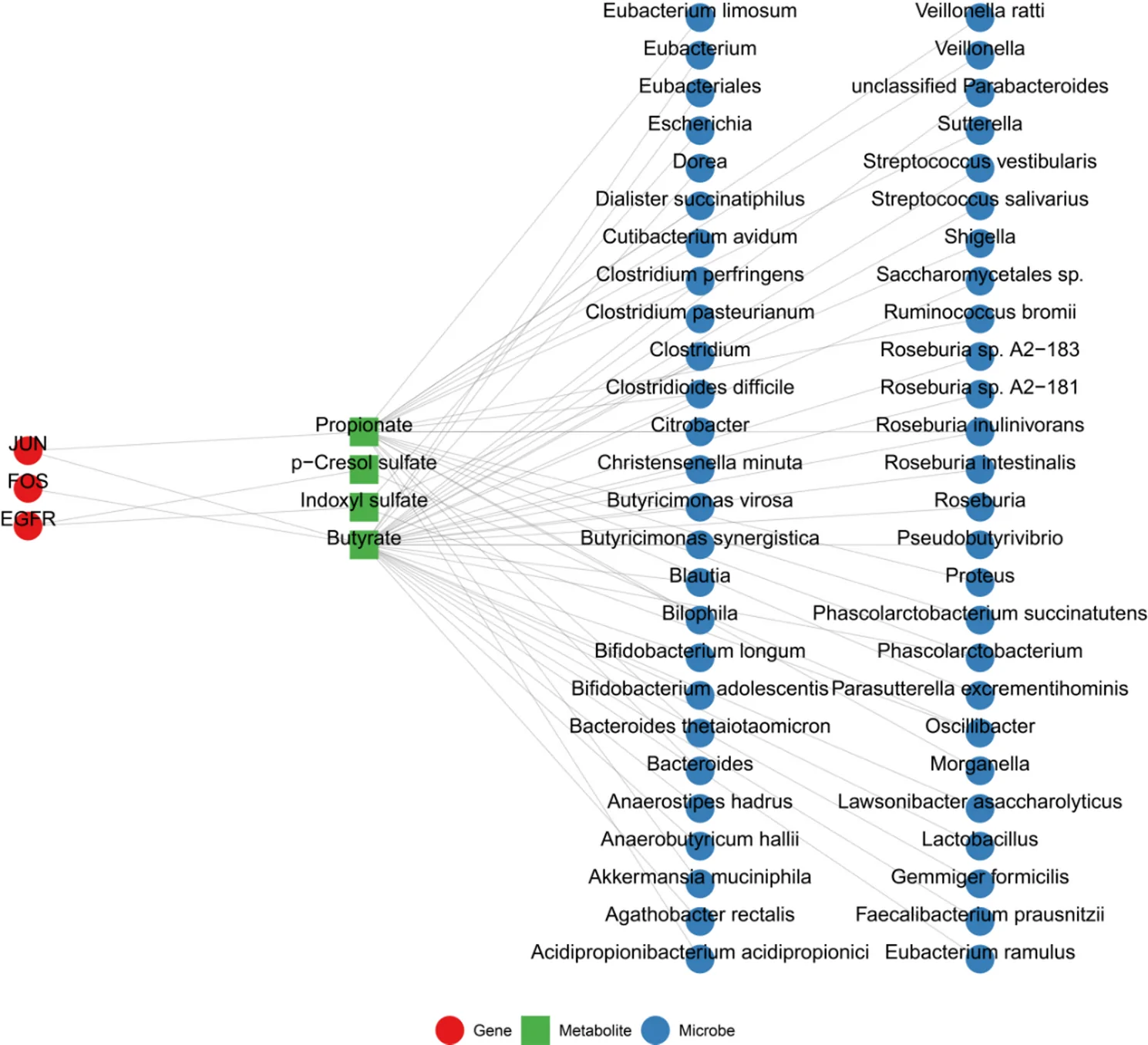
Interaction network of hub genes, the gut microbiota, and metabolites

To elucidate the potential regulatory mechanisms linking the identified hub genes with the gut microenvironment, we utilised the gutMGene database to construct a multi-omics interaction network. Among the seven candidate genes, only three (JUN, FOS, and EGFR) exhibited significant associations with the gut microbiota and metabolites and were thus included in the final network construction (Fig. 2).

As illustrated in the network, these three genes serve as central nodes connecting distinct microbial communities and metabolic factors. Specifically, network analysis revealed complex interconnections between these genes and specific bacterial taxa (Citrobacter, Clostridium, and Faecalibacterium) as well as key metabolites (propionate, p-cresol sulfate, indoxyl sulfate, and butyrate). Notably, the network includes both proinflammatory metabolites and opportunistic pathogens in addition to beneficial SCFA-producing bacteria, suggesting a multifaceted interplay between host genetic factors and the gut microenvironment. This network indicates that JUN, FOS, and EGFR may contribute to RA pathogenesis through the integration of signals from the gut microbiome composition and metabolic homeostasis.

### Construction of a risk scoring system and functional characterisation of high- and low-score groups

**Fig. 3:**
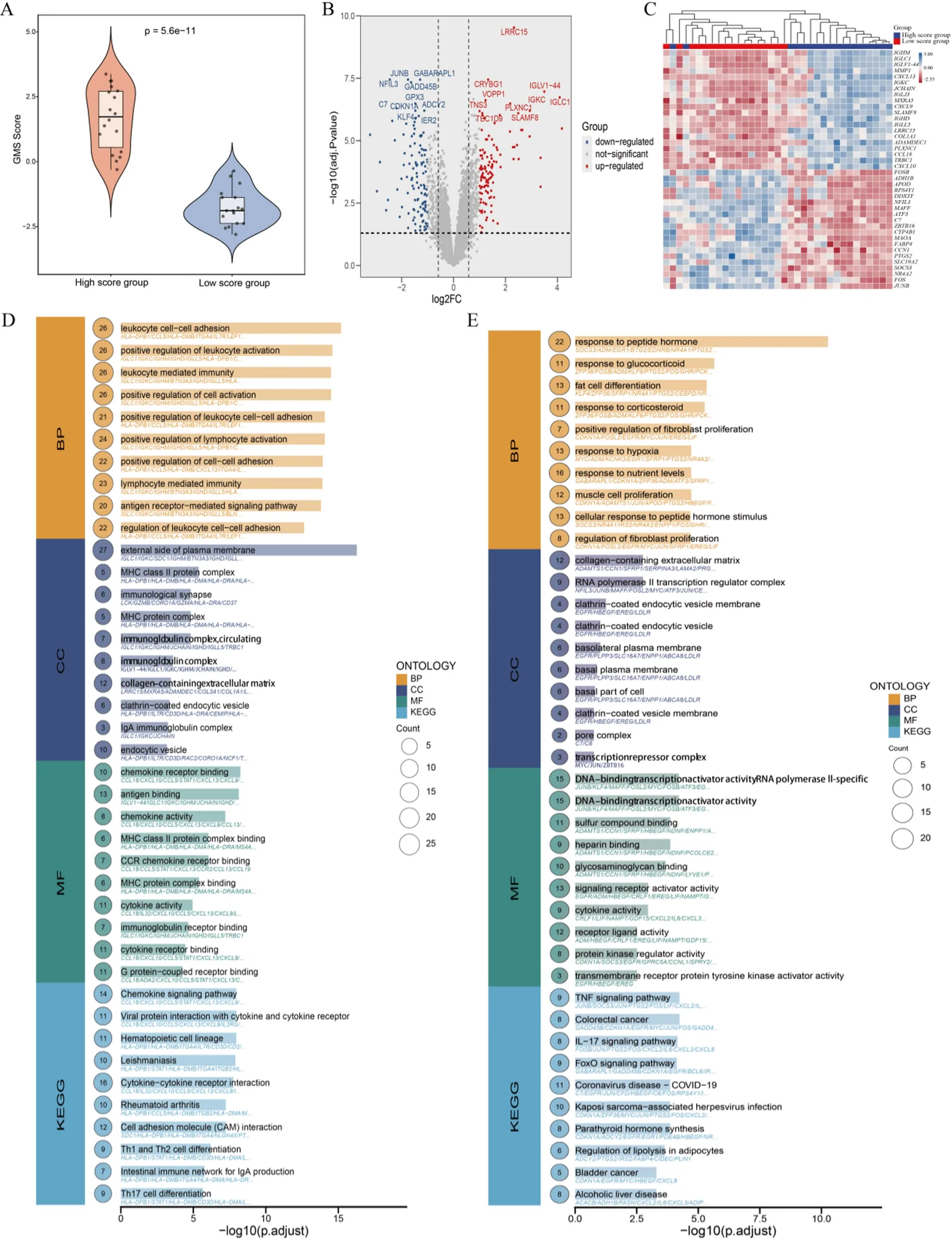
Identification and functional characterisation of two distinct RA subtypes. **A **Comparison of the GMS between the high-score group and low-score group. **B** Volcano plot displaying the DEGs between the two subtypes. **C** Heatmap showing the expression patterns of the DEGs in the high-score and low-score groups. **D** Functional enrichment analysis of the significantly upregulated genes in the high-score group. **E** Functional enrichment analysis of the significantly downregulated genes in the low-score group

To further investigate the biological implications of the identified 7 hub genes, we calculated a GMS for each patient with RA using PCA. The PC1 loading values for FOS, JUN, IDO1, EGFR, ABCC4, FADS3, and PEMT were − 0.3652, − 0.4157, 0.3457, − 0.4031, 0.4279, − 0.3574, and − 0.3179, respectively. On the basis of the median GMS, the cohort was stratified into a high-score group and a low-score group. The GMS was significantly different between the two subtypes (*p* = 5.6e-11) (Fig. 3A). We then performed differential expression analysis to explore the transcriptional variations between these two subtypes. A total of 713 DEGs were identified, with 404 upregulated and 309 downregulated genes in the high-score group compared with the low-score group. Volcano plots illustrated the top 10 most significantly up- and downregulated genes (Fig. 3B). The heatmap clearly distinguished the expression profiles of these DEGs between the two subtypes (Fig. 3C).

To understand the underlying biological mechanisms, GO and KEGG enrichment analyses were conducted separately for the upregulated and downregulated genes. Specifically, the genes upregulated in the high-GMS subgroup were enriched primarily in immune activation-related biological processes, such as leukocyte and lymphocyte activation, T-cell activation, and inflammatory responses. KEGG pathway analysis further revealed significant associations with immune-inflammatory pathways, including those involved in rheumatoid arthritis and Th17 cell differentiation. These findings suggest that the high-GMS subtype exhibits adaptive immune activation characterised by robust adaptive immune responses. In contrast, the enrichment pattern of the low-GMS subtype was distinct. Notably, KEGG analysis revealed that the low-GMS subtype highly expressed TNF signalling and IL-17 signalling pathways, along with FoxO signalling, colorectal cancer, COVID-19, alcoholic liver disease, and the regulation of lipolysis in adipocytes. GO analysis further revealed enrichment in responses to peptide and glucocorticoid hormones, fat cell differentiation, hypoxia, fibroblast proliferation, and collagen-containing extracellular matrix organisation. Collectively, these findings indicate that the low-GMS subtype exhibits a complex phenotype characterised by the concurrent activation of TNF/IL-17 inflammatory pathways, metabolic dysregulation, and cellular stress responses (Fig. 3D and E). Accordingly, we defined the high- and low-score subtypes as an adaptive immune activation subtype and an inflammatory-metabolic composite subtype, respectively.

### Immune infiltration landscape and gene‒immune interaction network

**Fig. 4:**
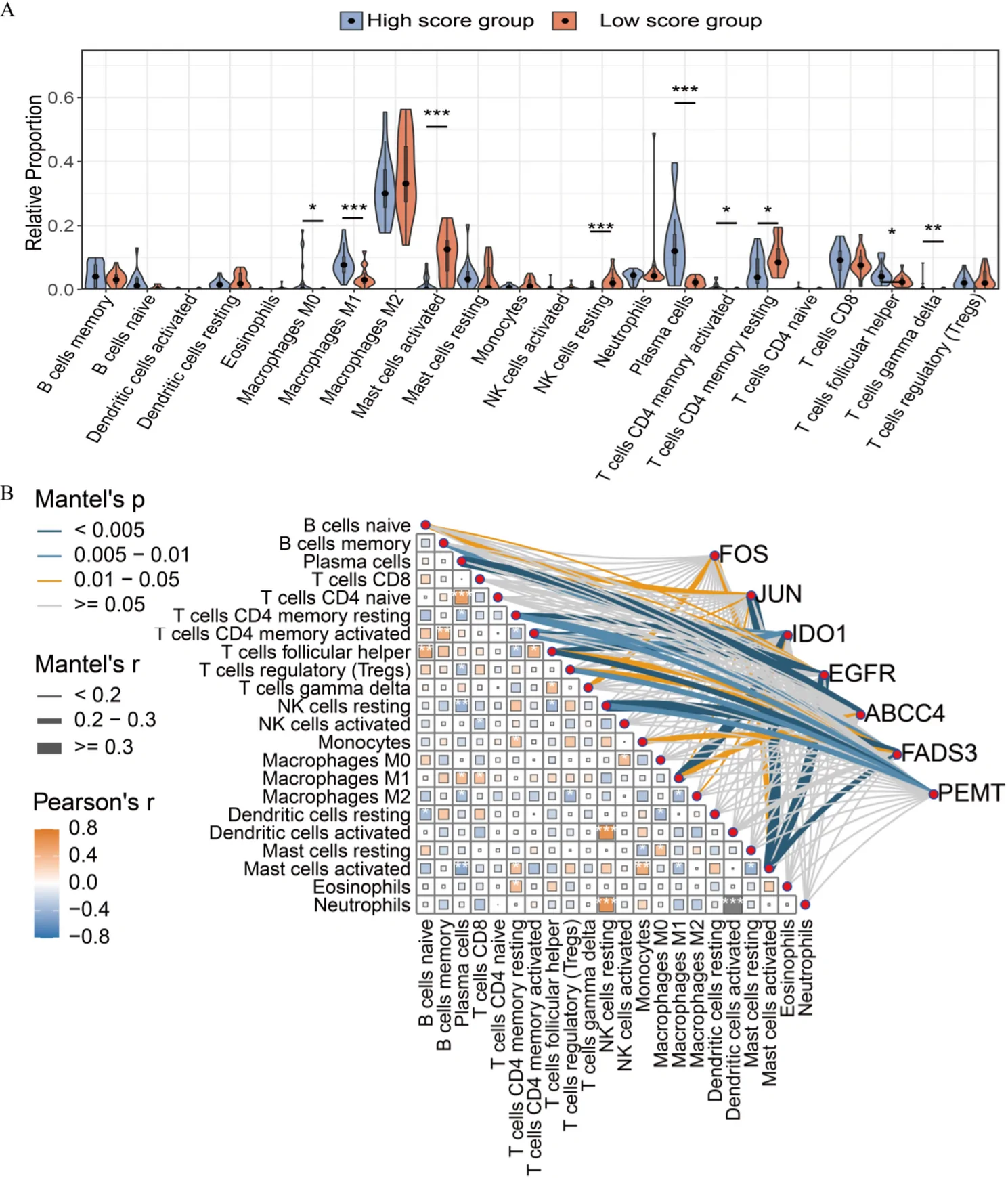
Immune infiltration landscape and gene‒immune interaction network. **A** Comparison of the relative proportions of 22 immune cell types between the high-score group and low-score group. *p < 0.05, **p < 0.01, ***p < 0.001. **B** Correlation network between the seven hub genes and immune cells

To characterise the immune microenvironment associated with the hub genes, we first compared the infiltration levels of 22 immune cell types between the two subtypes. As shown in Fig. 4A, the high-score group exhibited a significantly greater abundance of proinflammatory cells, particularly M1 macrophages and activated CD4 + memory T cells (*p* < 0.05), and a marked reduction in the number of resting NK cells and naive CD4 + T cells. These findings align with the previously identified enrichment of inflammatory pathways, suggesting an activated immune state in the high-score group. To further elucidate the specific regulatory roles of our candidate genes, we constructed a correlation network linking the seven hub genes to immune infiltrates (Fig. 4B). Notably, JUN, FOS, and EGFR were positively correlated with proinflammatory populations enriched in the high-score group, including M1 macrophages and activated dendritic cells. IDO1 expression also displayed a similar correlation pattern with these cell types. In contrast, ABCC4, FADS3, and PEMT exhibited distinct correlation patterns, which were primarily associated with resting immune cell subsets, such as resting dendritic cells and resting mast cells. Collectively, these findings suggest that JUN, FOS, and EGFR may play important roles in shaping the proinflammatory immune landscape of the high-score group.

### Value of the GMS for predicting the therapeutic response in patients with RA

**Fig. 5:**
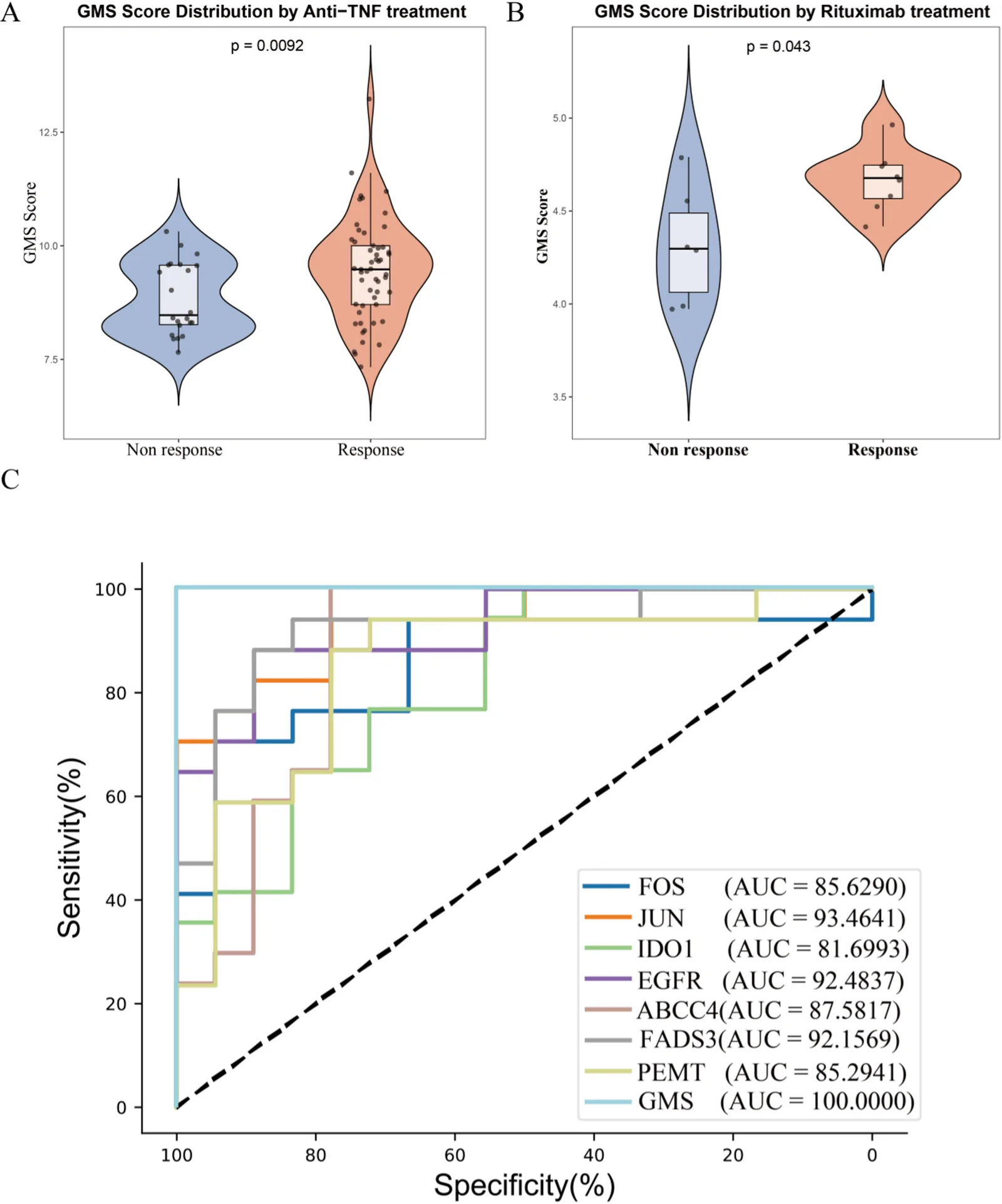
GMS as a predictive biomarker for therapeutic response in patients with RA. **A**, **B** Violin plots illustrating the distribution of GMS in patients with RA stratified by treatment response. Patients were treated with either anti-TNF therapy (**A**) or rituximab therapy (**B**). **C** ROC curve analysis evaluating the predictive accuracy of the seven hub genes and the composite GMS

To investigate whether the GMS could serve as a biomarker for predicting therapeutic efficacy, we analysed its distribution in patients with RA treated with anti-TNF and rituximab. As illustrated in Fig. 5A and B, the GMS was significantly greater in the response group than in the nonresponse group for both treatment modalities (anti-TNF-α: *p* = 0.0092; rituximab: *p* = 0.043). These findings indicate that patients with an elevated GMS are more likely to benefit from these interventions (Fig. 5C).

Furthermore, we performed ROC curve analysis to evaluate the predictive accuracy of the seven hub genes and the GMS (Fig. 5C). The analysis revealed that individual genes, including JUN (AUC = 0.935), FADS3 (AUC = 0.922), and EGFR (AUC = 0.924), exhibited favourable predictive potential. Notably, the GMS demonstrated outstanding discriminative performance in this validation cohort, with an AUC of 1.000. Nevertheless, we recognise that this perfect discrimination may carry a risk of overfitting, and its predictive stability warrants further calibration and validation in more diverse real-world populations.

### Single-cell transcriptome landscape and in silico knockout analysis of hub genes

**Fig. 6:**
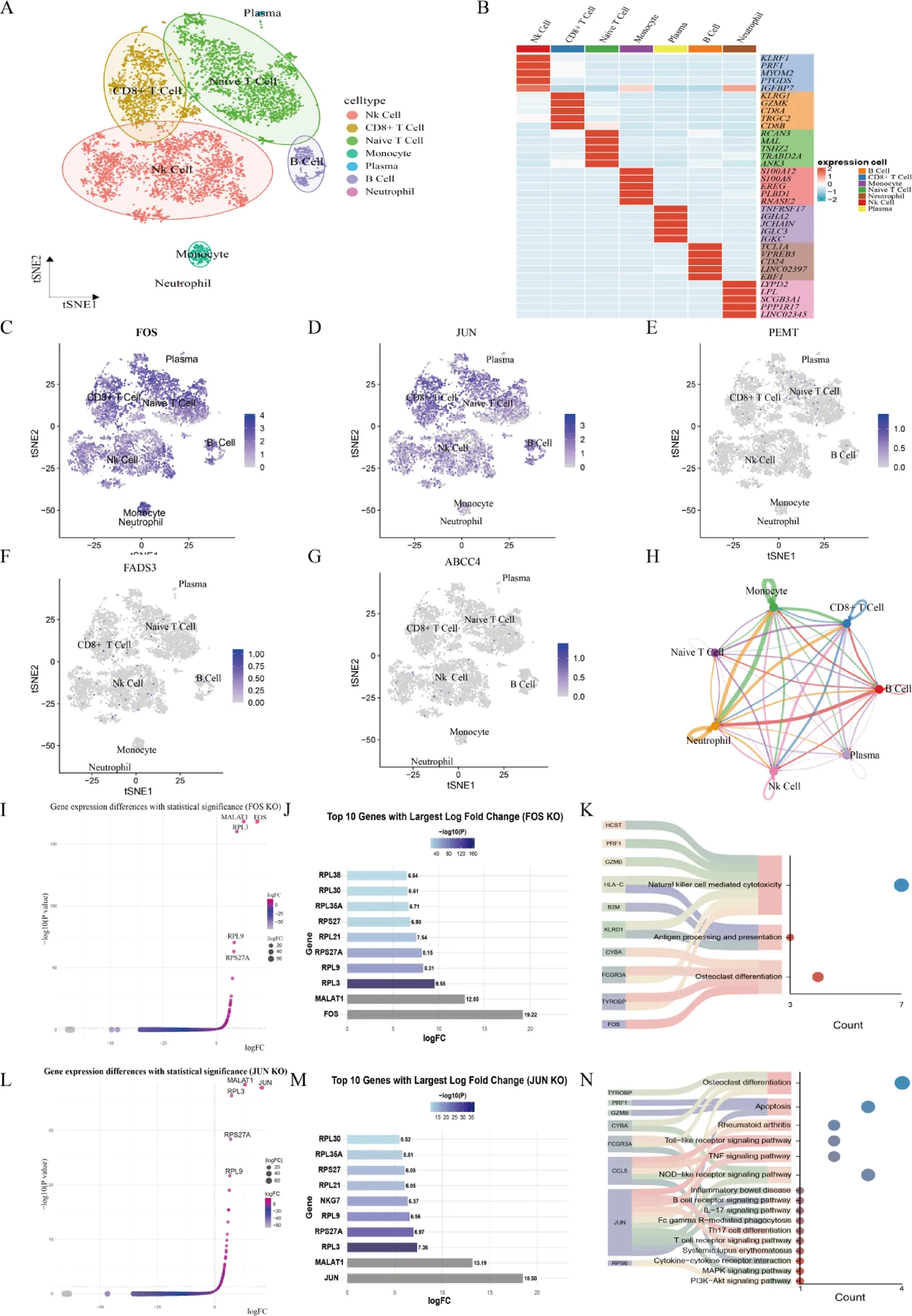
Single-cell transcriptomic landscape and in silico knockout analysis. **A** t-SNE plot showing the distribution of major immune cell types. **B** Heatmap of cell type-specific marker gene expression. **C**–**G** Feature plots showing the expression distribution of the hub genes. **H** Cell–cell communication network. **I** Volcano plot of differential gene expression following FOS knockout. **J** The top 10 genes with the greatest log fold changes in expression following a FOS knockout. **K** Functional enrichment analysis of FOS-regulated genes. **L** Volcano plot of differential gene expression following JUN knockout. **M** The top 10 genes with the greatest log fold change in expression after JUN knockout. **N** Functional enrichment analysis of JUN-regulated genes

To characterise the cellular landscape of RA, we analysed a scRNA-seq dataset and identified seven distinct cell populations: NK cells, CD8 + T cells, naive T cells, monocytes, neutrophils, plasma cells, and B cells (Fig. 6A). Furthermore, a heatmap of marker gene expression confirmed the distinct identity of each cell cluster (Fig. 6B). We subsequently mapped the expression of the hub genes across these cell types. As shown in the feature plots, FOS and JUN were widely expressed and particularly enriched in T cells and NK cells (Fig. 6C and D), whereas genes such as PEMT, FADS3, and ABCC4 exhibited more restricted expression patterns.

Cell‒cell communication analysis revealed intricate interaction networks within the RA microenvironment (Fig. 6H). The interaction strength heatmap demonstrated that monocytes and neutrophils acted as the primary senders of intercellular signals, exhibiting strong outgoing communication intensity to other cell types, particularly towards T cells and B cells. These findings suggest that myeloid cells play a dominant role in driving the inflammatory cascade within the RA synovium.

To further elucidate the regulatory mechanisms of the top hub genes, we performed in silico knockout analyses targeting FOS and JUN. With respect to the FOS knockout (Fig. 6I and K), the simulation identified a set of significantly downregulated genes, with MALAT1, RPL3, and FOS itself exhibiting the greatest log_2_ FC. Functional enrichment analysis of these genes revealed that they were significantly enriched in pathways related to natural killer cell-mediated cytotoxicity, antigen processing and presentation, and, notably, osteoclast differentiation. These findings indicate that FOS may contribute to RA pathology by regulating bone erosion and immune recognition processes. Similarly, the in silico perturbation of JUN (Fig. 6L and N) resulted in significant transcriptional alterations, with MALAT1, JUN, and ribosomal proteins (RPL3, RPS27A) being the most significantly affected genes. The enrichment analysis revealed that these DEGs were primarily associated with osteoclast differentiation, apoptosis, and key inflammatory signalling pathways, including the TNF signalling pathway, Toll-like receptor signalling pathway, and MAPK signalling pathway. These findings suggest that JUN acts as a central regulator of inflammation and cell survival in the RA microenvironment, potentially through the MAPK and TNF axes.

### Molecular docking visualisation

**Fig. 7:**
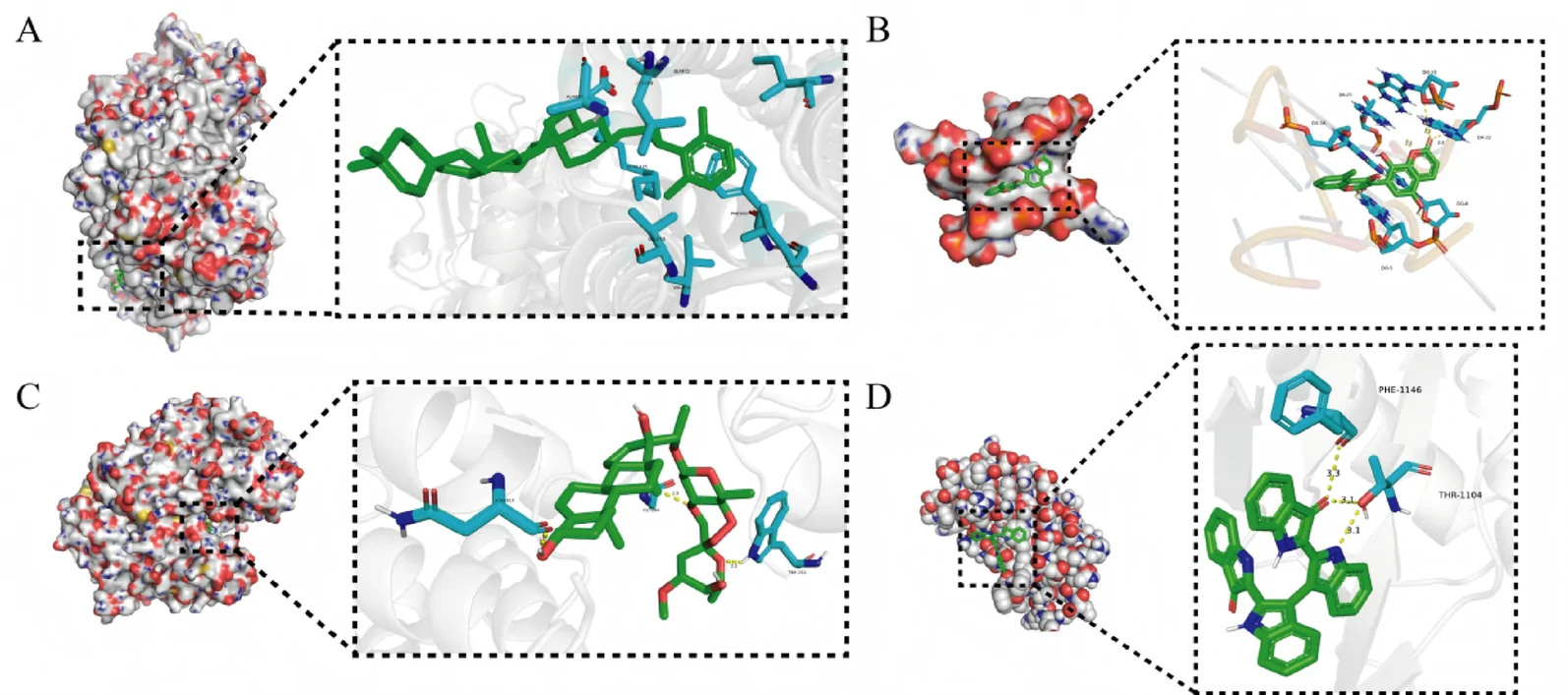
**A** Three-dimensional structural analysis of ABCC4 bound to the optimal small molecule. **B** Three-dimensional structural analysis of EGFR bound to the optimal small molecule. **C** Three-dimensional structural analysis of IDO1 bound to the optimal small molecule. **D** Three-dimensional structural analysis of PEMT bound to the optimal small molecule

The protein structures of ABCC4, IDO1, EGFR, and PEMT (PDB IDs: 8XOK, 5ETW, 8JFQ, and 6BSB) were downloaded from the PDB database. A total of 502 Chinese herbal medicines, encompassing 2,583 active small molecules, were included from the TCMSP database. High-throughput molecular docking revealed that the active compounds MOL008559, MOL005675, MOL003199, and MOL011100 exhibited strong binding affinities towards ABCC4, IDO1, EGFR, and PEMT, respectively, with binding energies of − 12.5 kcal/mol, − 10.8 kcal/mol, − 10.3 kcal/mol, and − 8.8 kcal/mol, respectively. The corresponding Chinese herbal medicines include Tribulus terrestris (Tribuli Fructus), Periploca sepium (Cortex Periplocae), Tripterygium wilfordii, Polygonum tinctorium (Polygoni Tinctorii Folium), and Indigo Naturalis. The visualisation results are presented in Fig. 7.

## Discussion

This study systematically established a GMS for molecular subtyping and therapeutic response prediction in patients with RA and validated the core regulatory mechanism of the gut–joint axis via single-cell transcriptomics and in silico knockout. Our findings provide a multi-omics integrated strategy for precision medicine in RA and identify FOS/JUN as critical effector genes that link gut microenvironmental signals to joint inflammation and bone destruction.

The gut–joint axis has emerged as a critical factor in the pathogenesis of RA, in which gut dysbiosis is closely intertwined with immune dysregulation. The gut microbiome not only participates in digestion and barrier maintenance but also affects systemic immunity through mechanisms such as microbial translocation, metabolite signalling, and immune cell trafficking (Kuhn et al. 2025). In this study, we identified seven hub genes that are significantly dysregulated in RA and are annotated in the gutMGene database as targets of gut microbial metabolites, suggesting their potential role as molecular bridges linking gut-derived signals to host pathology. FOS and JUN form the AP-1 transcription factor complex as a dimeric unit, which serves as a key signalling hub through which cells respond to diverse extracellular stimuli and activate genes involved in proliferation, differentiation, apoptosis, and tissue repair (Sarate et al. 2024). Notably, functional somatic mutations have been identified in the coding regions of FOS and JUN in the synovial tissues of patients with RA, which may further increase AP-1 transcriptional activity and upregulate MMP-1 expression, suggesting that the pathogenic potential of this complex may be amplified by genetic alterations in a subset of patients (Huber et al. 2020). In articular cartilage, the excessive activation of AP-1 induced by *Micrococcus luteus* G18 disrupts chondrocyte homeostasis and promotes extracellular matrix degradation, thereby accelerating OA progression (Chen et al. 2026). Moreover, immune–metabolic crosstalk may represent an important link within this regulatory axis. Indoleamine 2,3-dioxygenase 1 (IDO1), a key rate-limiting enzyme in the tryptophan–kynurenine metabolic pathway, functions as an important metabolic sensor that is responsive to gut microbial signals. Increased IDO1-mediated tryptophan metabolism has been associated with better responses to conventional synthetic disease-modifying antirheumatic drugs (csDMARDs), lower inflammatory activity, and improved clinical outcomes in patients with RA (Jian et al. 2026). Furthermore, pharmacological studies have shown that decitabine can selectively enter activated effector T cells through the ENT1 transporter, inducing the apoptosis of pathogenic Th1/Th17 cells and promoting the expansion of regulatory T cells. The sustained therapeutic effects of this approach depend on IDO1 activity, suggesting that combining T-cell immune reconstitution with IDO1-related metabolic modulation may represent a potential strategy for achieving durable remission in patients with RA (Huang et al. 2021). In addition, lipid- and membrane-related signalling may contribute to joint homeostasis and tissue damage. Activation of the EGFR signalling pathway can delay OA progression by promoting chondrocyte proliferation and maintaining cartilage homeostasis (Wei et al. 2021). ABCC4 is highly expressed in CD142 + synovial fibroblasts and promotes their invasive phenotype and meniscal damage by exporting cAMP and suppressing cAMP/PKA signalling, whereas blocking ABCC4 expression alleviates meniscal destruction (Sun et al. 2025a, b). Collectively, these findings suggest that the identified hub genes may play critical roles in RA through the integration of inflammatory, metabolic, and tissue-remodelling signals, thereby contributing to persistent joint inflammation and tissue destruction. These genes may therefore represent key molecular nodes linking gut-derived signals to joint pathology and serve as potential therapeutic targets in RA.

By integrating gut microbiota–metabolite–host genes, we constructed a GMS scoring system to precisely stratify the functional subtypes of RA. This study systematically elucidates the heterogeneity of the immune activation states and metabolic regulatory patterns of patients with RA while further validating the feasibility and scientific robustness of using the GMS as a biomarker for predicting treatment response.

The high-GMS subtype is dominated by adaptive immune activation, whereas the low-GMS subtype presents a complex phenotype characterised by concurrent TNF/IL-17 inflammatory activation, metabolic dysregulation, and cellular stress responses. These two subtypes display markedly divergent characteristics at the level of signalling pathway regulation. Accumulating evidence has demonstrated that aberrant Th17 cell proliferation, the massive release of proinflammatory cytokines, and the excessive activation of myeloid immune cells are core drivers of the initiation and progression of synovial inflammation in patients with RA, in which a persistent inflammatory microenvironment exacerbates synovial hyperplasia, cartilage damage, and bone destruction (Yang et al. 2019; Maeda et al. 2022). Dysregulation of metabolic homeostasis not only disrupts the body’s immune regulatory network but also alters metabolite levels within the synovial microenvironment, thereby weakening responsiveness to targeted immunotherapies. By simultaneously capturing immune and metabolic regulatory features, the GMS explains RA heterogeneity at the molecular functional level. This distinguishes it from previous subtyping models that focused solely on immune dysregulation and overcomes the limitations of traditional classification systems that rely heavily on clinical indices, serological markers, and single immune parameters, thereby providing new theoretical support for deciphering the heterogeneity of RA pathogenesis and advancing precision medicine (Zhao et al. 2021).

Furthermore, the core hub genes identified in this study, including FOS and JUN, were significantly positively correlated with proinflammatory immune cell subsets. As classical regulators of inflammation, the FOS/JUN complex participates in the transcriptional regulation of inflammatory signalling pathways such as NF-κB and MAPK, thereby mediating the translation of gut microbial signals into host local inflammation (Gong et al. 2022). In contrast, metabolism-related genes such as ABCC4, FADS3, and PEMT are associated primarily with resting immune cell subsets and are involved in lipid metabolism, substance transport, and metabolic homeostasis, further suggesting deep coupling between metabolic regulatory pathways and the maintenance of immune homeostasis (Sun et al. 2025a, b; Dai et al. 2025). Collectively, these results indicate that key hub genes can bridge gut microecological signals, host metabolic regulation, and immune cell differentiation to jointly drive the formation of distinct functional RA subtypes, further validating the efficacy of the GMS model in comprehensively evaluating the immune microenvironment status of patients with RA.

At the clinical translation level, our study revealed that compared with the low-GMS subtype, the high-GMS subtype resulted in significantly better treatment responses. Consistently, GMS levels were significantly higher in responders than in nonresponders. ROC curve analysis further demonstrated the good predictive performance of the GMS in this cohort. However, given the limited sample size and single-cohort design, this near-perfect discriminatory performance should be interpreted with caution to avoid overly optimistic conclusions and the potential risk of overfitting. Notably, we observed an apparent discrepancy in that the low-GMS subtype showed significant enrichment of TNF and IL-17 signalling pathways, whereas patients with a high GMS exhibited a more favourable response to anti-TNF therapy. We speculate that these seemingly paradoxical findings may reflect distinct dimensions of RA heterogeneity. Functional enrichment analysis primarily captures local inflammatory and tissue-destructive programs at the tissue level, whereas the GMS is derived mainly from peripheral blood transcriptomic features and may therefore more accurately reflect the systemic immune state and therapeutic response potential of individual patients (Lee et al. 2020). Specifically, the concurrent activation of TNF and IL-17 signalling in the low-GMS subtype may indicate a more complex inflammatory and metabolic stress state, which has been associated with primary resistance to TNF blockade (Alzabin et al. 2012; D.Y. Chen et al. 2011). In contrast, the high-GMS subtype is characterised by adaptive immune activation, an immunological phenotype that has been widely associated with favourable responses to biologic therapies (Humby et al. 2021; Rivellese et al. 2022; Farutin et al. 2019; Tao et al. 2021; Tanino et al. 2009). Taken together, these findings highlight distinct but complementary dimensions of RA heterogeneity and suggest that the GMS may serve as a promising quantitative tool for individualised treatment selection. Further validation in larger, prospective, multicentre cohorts is warranted.

We performed large-scale molecular docking of the four targets against all the small-molecule constituents available in the TCMSP database and, for each target, identified the compound that exhibited the lowest binding energy. Specifically, strong binding affinities were observed between the active compounds MOL008559, MOL005675, MOL003199, and MOL011100 and their respective target proteins, including ABCC4, IDO1, EGFR, and PEMT, with binding energies of − 12.5 kcal/mol, − 10.8 kcal/mol, − 10.3 kcal/mol, and − 8.8 kcal/mol, respectively. These results provide computational evidence supporting the potential binding interactions between TCMSP-derived small-molecule constituents and the identified hub proteins, thereby highlighting their possible pharmacological relevance.

Despite the notable findings of this study, several limitations should be acknowledged. First, our analyses were primarily based on public transcriptomic datasets and lacked validation from prospective, large-scale, multicentre clinical cohorts. Therefore, the clinical predictive value and robustness of the GMS require further verification. Second, our evaluation of treatment response was limited to two mainstream biologic agents; the predictive value of the GMS for other therapeutic modalities, including metabolism-targeting drugs and novel immunosuppressants, remains to be determined.

## Conclusion

In conclusion, by constructing a scoring system based on the gut microbiota–metabolite–host gene axis, this study successfully achieved the functional subtyping of RA into an adaptive immune activation subtype and an inflammatory–metabolic composite subtype. This study provides in-depth insights into the divergent immune microenvironments and molecular regulatory mechanisms across subtypes while also validating the clinical utility of the GMS in predicting biological therapy responses. Future research should focus on prospective clinical cohort studies to further validate the GMS, elucidate the specific regulatory mechanisms driving each subtype, and explore composite therapeutic strategies combining immune-targeted and metabolism-modulating approaches. Such efforts will provide a solid theoretical and practical foundation for overcoming RA heterogeneity and advancing precision medicine.

## Data Availability

Not applicable.
